# Use of NT‐proBNP for the screening, diagnosis and risk‐stratification of left ventricular dysfunction

**DOI:** 10.1111/dom.16388

**Published:** 2025-04-09

**Authors:** Pardeep S. Jhund

**Affiliations:** ^1^ BHF Glasgow Cardiovascular Research Centre, School of Cardiovascular and Metabolic Health University of Glasgow Glasgow UK

**Keywords:** heart failure, natriuretic peptides, screening, ventricular dysfunction

## Abstract

Heart failure (HF) is a major health problem, and preventing the onset of heart failure could have large cost implications for healthcare systems globally. Screening for heart failure and its precursor, left ventricular dysfunction, could allow patients to receive therapies shown to reduce the risk of incident heart failure, such as ACE inhibitors and beta blockers. Using echocardiography to screen patients is costly. Natriuretic peptides could be used to screen populations for asymptomatic left ventricular function. However, natriuretic peptide levels vary by age, sex and presence of comorbidities such as atrial fibrillation and kidney disease. Using one threshold value in a large population may impair the sensitivity and specificity of such an approach, but prior studies in community‐based adults suggest that this is a feasible strategy. A higher yield strategy would be to screen high‐risk patients, such as those with diabetes mellitus, and current guidelines for the management of diabetes suggest using natriuretic peptides to screen patients for unrecognised heart failure. Natriuretic peptides can also help ascertain the risk of future cardiovascular events and deaths in patients with diabetes. Natriuretic peptides have established themselves as a central part of the definition of heart failure. However, more work needs to be done to determine the optimal age, sex and body weight‐based thresholds, as well as thresholds for those with comorbidities like atrial fibrillation and chronic kidney disease. These are needed to determine and optimise the sensitivity and specificity of natriuretic peptides in the diagnosis of heart failure. Clinicians should use guideline‐recommended thresholds to diagnose HF with natriuretic peptides but consider factors that influence levels, such as age, kidney function, etc. It is yet unclear if natriuretic peptides can be used to guide the management of patients with heart failure.

## 1 INTRODUCTION

**FIGURE 1:**
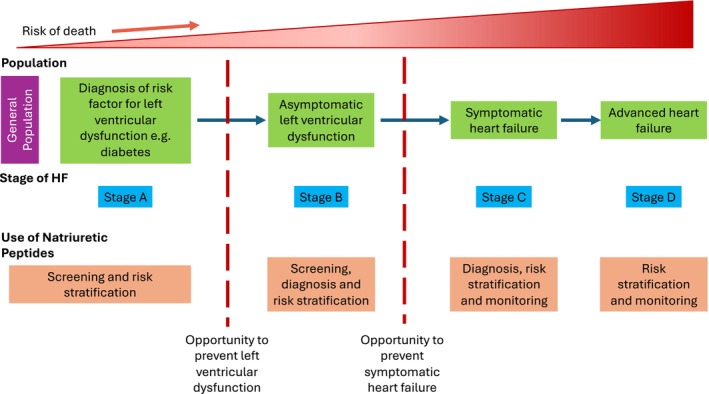
Stages of heart failure from the general population through to risk factors and end‐stage heart failure. Opportunities to utilise natriuretic peptides to screen for asymptomatic disease and determine risk.

Heart failure affects around 64 million people worldwide.^1^ Once a person develops heart failure, the prognosis is similar to many cancers.^2^ While the treatment of heart failure has made many advances over the last few decades, it is imperative that if we are to continue to reduce the burden of heart failure on patients and healthcare systems, we need to try and prevent heart failure. The syndrome of heart failure consists of signs and symptoms of congestion that are caused by neurohormonal activation.^3^ This commonly results from left ventricular dysfunction, which can be due to systolic dysfunction of the left ventricle or other abnormalities of cardiac function such as diastolic dysfunction of the left ventricle. Left ventricular dysfunction can occur as a result of numerous conditions and may be asymptomatic. Therefore, the detection of ventricular dysfunction represents an important part of the pathway by which we can prevent the development of heart failure (Figure 1). In the current classification of heart failure, the presence of risk factors is called Stage A, the presence of cardiac abnormalities increasing the risk of heart failure is called Stage B. Overt heart failure places a patient in Stage C, and advanced heart failure (requiring specialist intervention such as mechanical circulatory support or transplantation) is Stage D (Figure 1). The aim of prevention of heart failure is to prevent patients in Stage A from progressing to Stage B and patients in Stage B from progressing to Stage C, where their risk of morbidity and mortality increases greatly. One of the major predictors of progressing from Stage B to C is the presence of left ventricular dysfunction. Detecting left ventricular dysfunction has been difficult as it is often asymptomatic. The need for echocardiography to look for left ventricular dysfunction is obviously problematic, as it is an expensive test (relatively speaking when compared to simple blood tests) and it requires specialist equipment and individuals who can perform the test and interpret it. Furthermore, it relies on a care team referring the patient for the investigation, and given that many patients with risk factors such as diabetes have unrecognised symptomatic heart failure, many also have unrecognised asymptomatic left ventricular dysfunction.^4^ Ideally, patients would undergo an easy‐to‐use test such as a blood test that would facilitate screening for left ventricular dysfunction, allowing therapy to be used to prevent the onset of heart failure. A randomised trial of the ACE inhibitor, enalapril, showed that it is possible to reduce the risk of developing heart failure in patients with asymptomatic dysfunction^5^ and a trial of a beta blocker, carvedilol, also showed that these drugs could be used to prevent morbidity and mortality in left ventricular dysfunction following myocardial infarction.^6^ Therefore, detecting left ventricular dysfunction would be an appropriate target of a screening programme. As noted, echocardiography would be an expensive method by which to screen individuals at scale. The natriuretic peptides have emerged as a potential test to screen for asymptomatic left ventricular dysfunction.

## 2 NATRIURETIC PEPTIDES

The natriuretic peptides were first identified as granules in atrial tissues.^7^, ^8^ Subsequently, de Bold et al. showed that injection of extracted myocardial tissue from the atria induced a natriuresis and diuresis in rats.^9^ Further studies determined the structure of a number of natriuretic peptides.^7^ It was found that increased ventricular preload and afterload, with consequent elevated wall stress, lead to the production of pre‐pro B‐type natriuretic peptide (BNP) which is cleaved to BNP and N‐terminal proBNP (NT‐proBNP). BNP causes natriuresis and vasodilation (NT‐proBNP is physiologically inactive) in an attempt to reduce the load on the ventricles detected by the wall stress. Both BNP and NT‐proBNP can be measured in the peripheral blood and are comparatively stable, unlike other natriuretic peptides.^7^ While BNP was previously measured, it has become supplanted in most clinical laboratories by NT‐proBNP. NT‐proBNP has a longer half‐life and is more stable at room temperature. Furthermore, the advent of neprilysin inhibitors in clinical practice for the treatment of heart failure (these prevent the breakdown of BNP) makes NT‐proBNP a more reliable marker of natriuretic peptide levels so that BNP has gradually become less commonly measured.

It is well recognised that raised natriuretic peptides are found in patients with left‐ventricular dysfunction. The degree of elevation of natriuretic peptides is related to the degree of ventricular dysfunction and changes in corresponding invasive haemodynamic measures. However, given the criteria for a successful screening programme, there is interest in using natriuretic peptides as a screening test for left ventricular dysfunction.

## 3 USE OF NATRIURETIC PEPTIDES IN THE GENERAL POPULATION

Before undertaking any screening programme, a number of factors must be considered. The criteria for a population screening programme are given in Table 1.^10^ The most important criteria of a screening test have been well defined. The test should be simple, safe and precise, well validated, calibrated to the population being screened and when the disease of interest is found, there should be an effective intervention for patients identified through the screening process. The programme itself should be backed by evidence, and participation in the programme by individuals should outweigh any potential harms from over‐ or underdiagnosis or overtreatment. Finally, the programme should be cost‐effective.

**TABLE 1:** Classical screening criteria of Wilson and Jungner.

The condition being screened for should be an important health problem.
2 There should be an accepted treatment for patients with the disease.
3 Facilities for diagnosis and treatment should be available.
4 There should be a recognised asymptomatic/latent or early symptomatic stage.
5 There should be a suitable test or examination for the condition.
6 The test should be acceptable to the population being screened.
7 The natural history of the condition, including development from asymptomatic /latent to disease, should be adequately understood.
8 There should be an agreed policy on which patients to treat.
9 The screening programmes should be cost‐effective, accounting for the cost of screening and therapy.
10 The process of screening should be a continuing process.

As noted above, left ventricular dysfunction precedes heart failure, which is a major public health concern, making this an important condition to detect. Several studies have shown that natriuretic peptides can detect asymptomatic left ventricular dysfunction in numerous populations.^11^, ^12^, ^13^, ^14^, ^15^, ^16^ One of the first of these was conducted in Glasgow in the West of Scotland^16^. In a random selection of participants aged 25 to 74 years from general practice lists, 1252 participants with interpretable electrocardiograms and echocardiograms with completed questionnaires, and blood samples were analysed; the resulting concentrations of NT‐proBNP and BNP were higher in patients with left ventricular systolic dysfunction compared to those without. The participants with symptomatic and asymptomatic left ventricular dysfunction both had raised natriuretic peptide concentrations. The sensitivity and specificity were good, but the tests had better sensitivity than specificity. The study was the first large‐scale study to suggest that population‐level, community screening for ventricular systolic dysfunction could be done and could reliably detect patients with left ventricular dysfunction. There have been a number of subsequent studies in a number of different populations again suggesting that natriuretic peptides could be used to screen for left ventricular dysfunction in the general population, but these have had varying results, most likely due to the population studied and design of the programme. A meta‐analysis of 24 community‐based studies suggested a pooled sensitivity of NT‐proBNP was 87% (95% confidence interval (CI) 73 to 94%) and a specificity of 84% (95% CI 55 to 96%).^17^ The studies included in this analysis used several thresholds of natriuretic peptide levels, leading to variations in the achieved sensitivity and specificity and consequently a high degree of heterogeneity between studies. Sensitivity and specificity are products of the underlying risk of the population studied, as well as the thresholds used for diagnosis. Better sensitivity and specificity were achieved in higher risk populations, such as the elderly. Further complicating this issue is that levels of natriuretic peptides are related to other risk factors. For example, left ventricular dysfunction is more common in the elderly, but the elderly also have a higher risk of developing ventricular dysfunction.^18^ In general, however, the authors concluded that a threshold for NT‐proBNP of 311 pg/mL had a sensitivity of 74% and specificity of 85% to detect left ventricular dysfunction. Other meta‐analyses have concluded that natriuretic peptide testing is useful but have also questioned the sensitivity and specificity, especially in the elderly.^19^ Given these issues, screening a general population is difficult, and it may be more prudent to increase sensitivity and specificity by a more targeted approach in higher risk populations.

This is what is currently done with common screening tests such as breast cancer screening using mammography or ultrasound for abdominal aortic aneurysms, which target certain age groups. Therefore, when considering which populations to screen, generally comorbidities with a high risk of developing left ventricular dysfunction have been studied.

## 4 USE OF NATRIURETIC PEPTIDES IN CORONARY ARTERY DISEASE

Myocardial damage as a result of myocardial infarction is one of the commonest reasons for an individual to develop left ventricular systolic dysfunction. Coronary artery disease is still one of the main aetiologies of heart failure worldwide. Therefore, screening patients with coronary artery disease who may or may not have had an overt myocardial infarction represents a population in whom screening may be more cost‐effective with better sensitivity and specificity than a general population. While there is evidence that natriuretic peptides are higher in those with myocardial ischaemia and coronary artery disease,^20^, ^21^ the use of natriuretic peptides to screen for left ventricular dysfunction has not been fully characterised. In a study of 815 patients with coronary heart disease and no heart failure, a NT‐proBNP of less than 100 pg/mL reduced the probability of left ventricular dysfunction being present from a pretest probability of 18% to a post‐test probability of 6%. A high NT‐proBNP of over 500 pg/mL increased the pretest probability from 18% to 47%, suggesting NT‐proBNP may be a better test to rule out left ventricular dysfunction than rule it in.^22^ There is a clear role for NT‐proBNP in the risk‐stratification of patients with acute and chronic coronary syndromes, with higher levels being related to poorer outcomes.^23^, ^24^ Although levels are high in the immediate aftermath of a myocardial infarction,^25^ levels measured around 4 days after the event provide additional prognostic information in addition to ejection fraction and other clinical variables.^26^ Higher levels of NT‐proBNP are associated with a higher risk of developing heart failure or mortality. Again, there is no clear consensus on what level of NT‐proBNP should be used to initiate therapy or if therapy should be up‐titrated in response to measurements of NT‐proBNP.

## 5 USE OF NATRIURETIC PEPTIDES IN DIABETES MELLITUS

In patients with diabetes mellitus, the prevalence of heart failure is high.^27^ Unfortunately, many patients with diabetes have unrecognised heart failure despite the presence of symptoms.^28^ Patients with diabetes who have heart failure are at much higher risk of hospitalisations and death than patients with heart failure who do not have diabetes. Therefore, such patients would be an ideal population in which to screen for left ventricular systolic dysfunction and prevent the onset of heart failure.^29^ Screening in a high‐risk population such as this could improve the cost‐effectiveness of any such screening programme. For example, the estimated number of patients who need to be submitted to echocardiography following a positive NT‐proBNP result to detect one patient with left ventricular systolic dysfunction would be 767 in the general population but only 12 in a population with either hypertension or changes on the electrocardiogram but no symptoms.^30^ Screening for left ventricular dysfunction and, in particular, symptomatic heart failure using natriuretic peptides has therefore been recommended by professional bodies for the management of diabetes and cardiovascular disease.^31^, ^32^ As noted above, many studies have utilised other markers such as an abnormal electrocardiogram or other risk factors for left ventricular dysfunction to further improve the diagnostic accuracy of natriuretic peptides as a screening tool. However, to date, there is no consensus on how these tests should be used to determine the sensitivity and specificity of the threshold of natriuretic peptide used to rule in or rule out a diagnosis. While it is unclear how best to combine other factors with NT‐proBNP in the setting of screening, for risk prediction in diabetes, NT‐proBNP alone can adequately risk stratify patients with diabetes for future cardiovascular risk, with other variables adding little to the risk prediction over the measurement of NT‐proBNP alone.^33^, ^34^

While most prior studies have assessed the use of natriuretic peptides to detect left ventricular dysfunction, given the clear relationship between natriuretic peptides and subsequent risk of cardiovascular outcomes, it is logical to test whether a strategy of testing NT‐proBNP may lead to better outcomes regardless of the presence of left ventricular dysfunction. If NT‐proBNP not only detects left ventricular dysfunction but also other individuals with diabetes at high risk of cardiovascular events, then perhaps treating all patients with diabetes and a raised natriuretic peptide level could lead to an improvement in outcomes. This hypothesis has been tested in a randomised controlled trial. In the PONTIAC (NT‐proBNP Selected PreventiOn of cardiac eveNts in a populaTion of dIabetic patients without A history of Cardiac disease) trial, 300 patients with type 2 diabetes and an elevated NT‐proBNP (>125 pg/mL) and free of known cardiac disease were randomised to NT‐proBNP guided medical therapy or standard care.^35^ Medications that were intensified according to the protocol were ACE inhibitors or angiotensin receptor blockers and beta blockers. The risk of cardiovascular hospitalisation or death at 2 years was reduced in the NT‐proBNP guided arm (hazard ratio 0.35 95% CI 0.13–0.98, *p* = 0.04). Given the promising results of this approach, despite the limited sample size, a larger trial is currently underway (clinicaltrials.gov NCT02817360).

## 6 USE OF NATRIURETIC PEPTIDES IN HEART FAILURE

**FIGURE 2:**
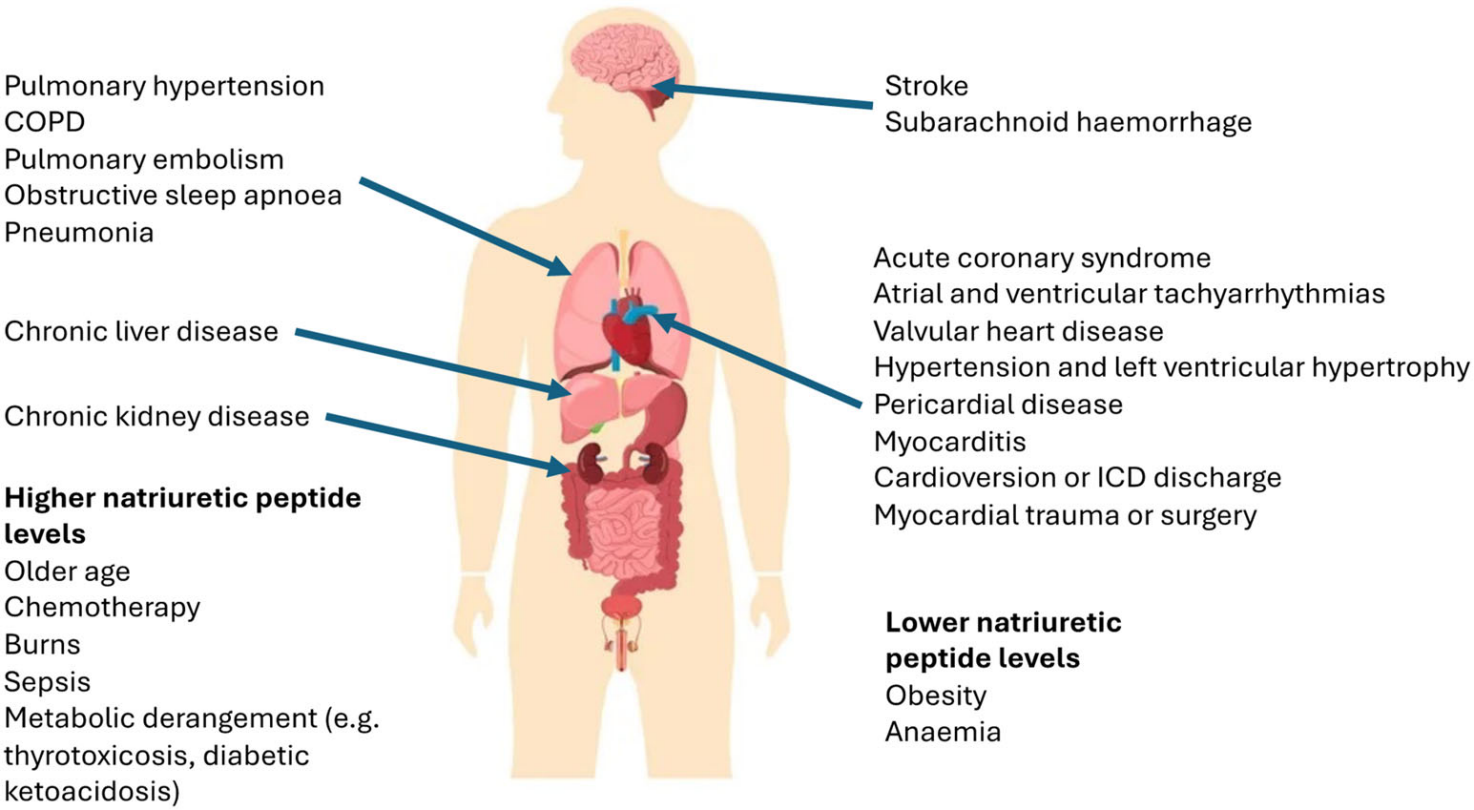
Comorbidities that can cause elevated natriuretic peptide levels and factors known to affect the interpretation of natriuretic peptide assays.

The area where most is known about the role of natriuretic peptides is in patients with heart failure. The use of natriuretic peptides for the diagnosis of heart failure is now common and standard practice.^3^ Given the overwhelming evidence that natriuretic peptide levels are raised in heart failure, the definition of heart failure incorporates raised natriuretic peptides as a key aspect of making the diagnosis. What is less clear, however, is the precise threshold to be used to rule in or rule out a diagnosis. Currently, the European Society of Cardiology guidelines use a value of BNP <35 pg/mL or NT‐proBNP <125 pg/mL to rule out heart failure in a non‐acute setting.^3^ In the acute setting, values of BNP <100 pg/mL or NT‐proBNP <300 pg/mL are suggested as appropriate thresholds to rule out heart failure. These thresholds apply to patients in sinus rhythm; however, as will be discussed later, a number of other conditions can raise natriuretic peptides and lower natriuretic peptides, and therefore care must be taken into account when interpreting high or low levels (Figure 2). Currently, guidelines do not give any guidance on how these levels should be adjusted according to factors known to influence natriuretic peptides and therefore alter the sensitivity and specificity of a cut‐off threshold value for ruling out or ruling in heart failure. Some suggestions for age and sex‐related cut‐offs have been made but are not universally used.^36^ The one comorbidity where there is more consensus about adjusting the cut‐off is in the presence of atrial fibrillation. It is suggested that thresholds are increased by 50–100% in the presence of atrial fibrillation.^3^, ^36^ There is currently no evidence to support different cut‐offs according to whether heart failure with reduced or preserved ejection fraction is suspected.

Recently it has been suggested that natriuretic peptides can be used during the diagnosis of heart failure to identify particularly high‐risk individuals who would merit more expedited review and investigation.^36^ This is due to the recognition that higher natriuretic peptide levels at diagnosis are associated with a higher risk of hospitalisation or death.^37^, ^38^ As with the diagnosis of heart failure there is ample evidence that higher natriuretic peptides are associated with a higher risk of hospitalisations, cardiovascular death and all‐cause death in patients with prevalent heart failure.^39^, ^40^, ^41^ This relationship is seen across the spectrum of ejection fraction and rising levels of natriuretic peptides are associated with a poorer prognosis and falling levels with a better prognosis. Currently there is little evidence that any of the guideline‐recommended therapies should be initiated on the basis of natriuretic peptide levels. While natriuretic peptides have been used to enrich trial populations for risk of subsequent events and confirm the diagnosis of heart failure, guidelines do not recommend their use for selecting patients for therapy. They can be used to monitor risk but how to incorporate natriuretic peptide monitoring into clinical practice is yet unclear. There is some evidence that NT‐proBNP can be incorporated into an up‐titration algorithm for guideline‐recommended therapies in heart failure. In the STRONG‐HF (Safety, Tolerability, and Efficacy of Rapid Optimization, Helped by N‐Terminal Pro–Brain Natriuretic Peptide Testing of Heart Failure Therapies) trial, changes in NT‐proBNP were incorporated into an algorithm for the rapid up‐titration of guideline‐recommended therapies for heart failure following discharge from hospital.^42^ They were used in conjunction with standard physical examination (e.g., blood pressure, heart rate) and laboratory measures (e.g., serum potassium and kidney function) to help up‐titrate beta blockers, inhibitors of the renin‐angiotensin aldosterone system and loop diuretics. While this trial showed a benefit of rapid up‐titration the precise contribution of NT‐proBNP measures to the results is not clear as it is hard to dissect its contribution from the other aspects of the algorithm.

## 7 FACTORS INFLUENCING NATRIURETIC PEPTIDE LEVELS

A number of physiological characteristics and comorbidities are known to influence natriuretic peptide levels. It is important that these comorbidities and characteristics are taken into account when interpreting the results of any assay (Figure 2). In particular, a number of conditions may influence the threshold at which the diagnosis of heart failure is ruled out, as with atrial fibrillation. Other conditions may need to be considered and interpreted with less specific alterations in the thresholds. Natriuretic peptide levels rise as people age. In a population‐level analysis of over 18 000 participants in a prospective cohort study, the median NT‐proBNP level was 21 pg/mL in those male participants aged <30 years, rising to 281 pg/mL in those ≥80 years. In female participants, the respective levels were 51 pg/mL in those <30 years and 240 in those ≥80 years.^43^ These age‐ and sex‐related differences in what constitutes a normal level of NT‐proBNP have been observed in multiple studies.^18^, ^37^ Until recently, these have been generally not taken into account when determining cut‐offs for the diagnosis of heart failure, but recently efforts have been made to incorporate age into the thresholds used for the diagnosis of heart failure.^36^ It has been suggested that age is used to adjust the levels used for screening for left ventricular dysfunction in the community and for the diagnosis of heart failure in the outpatient setting and emergency department. Race also appears to modulate normal levels of natriuretic peptides.^44^ Similarly, chronic kidney disease is a common comorbidity in patients with cardiovascular disease or other diseases such as diabetes that place patients at risk of developing ventricular dysfunction. As estimated glomerular filtration rates fall, levels of natriuretic peptides increase. This can be due to further wall stress from fluid retention but also a reduction in the clearance of natriuretic peptides by the kidney. As with age, attempts have been made to integrate kidney function into the thresholds of NT‐proBNP used to diagnose heart failure, but less so in risk‐stratification or screening.^36^ While there are multiple causes of high natriuretic peptides, there are also important causes of low natriuretic peptides. Obesity is a growing health problem, and natriuretic peptides are cleared not only in the kidney or by neutral endopeptidases but also by receptors in tissues.^7^, ^45^ Adipose tissue highly expresses the natriuretic peptide clearance receptors‐C and therefore increases natriuretic peptide clearance, but there may also be a bi‐directional relationship with adiposity, with natriuretic peptides having lipolytic effects.^45^ Lowering thresholds for natriuretic peptides for the diagnosis of heart failure may improve the sensitivity or specificity of the test in those with obesity. It is also not clear how patterns of regional fat distribution in the body may alter the utility of natriuretic peptides and how this should be accounted for in any adjustment to diagnostic thresholds.^46^ These factors also alter the utility of natriuretic peptides for risk prediction, but the impact of this is often less, as multivariable models are frequently used to estimate risk and therefore these differences can be adjusted for. In screening and diagnosis, there is less use of multivariable models in the clinical environment, especially in acute settings, to adjust diagnostic thresholds. Integrating these differences in natriuretic peptide levels in the screening for left ventricular dysfunction or diagnosis of heart failure remains an issue that requires consensus.

## 8 VARIATION IN ASSAYS

A final, less discussed pitfall of using NT‐proBNP as a measure for screening or diagnosis is the inter‐assay variation in the results. Multiple comparisons have been performed with different assays and have consistently shown that there is variation between the assays in their performance.^47^, ^48^, ^49^ Any future screening programmes or use of biomarkers for risk‐stratification would need to specify the precise assay being used.

## 9 CONCLUSIONS

Natriuretic peptides are a useful tool for the diagnosis and risk‐stratification of patients with heart failure. The precise thresholds that have the optimal sensitivity and specificity for the detection of heart failure have to be refined in growing recognition that important factors such as age, sex and kidney function all influence the sensitivity and specificity of these cut‐offs. Currently defined thresholds outlined in guidelines for the diagnosis of HF should be used in clinical practice with recognition that other factors may influence levels of natriuretic peptides. These factors should be considered by clinicians when interpreting the results of natriuretic peptide levels. In the prevention of heart failure, natriuretic peptide levels are associated with the presence of left ventricular dysfunction in patients at risk of developing heart failure. It is possible to screen populations using natriuretic peptides for the presence of asymptomatic left ventricular dysfunction. However, as with the diagnosis of heart failure, developing suitably sensitive and precise thresholds that account for variables known to affect natriuretic peptide levels requires further work. In the interim, using natriuretic peptides in those individuals at highest risk of having left ventricular dysfunction or heart failure, such as those with diabetes, remains the best use of this biomarker as a screening tool.

## CONFLICT OF INTEREST STATEMENT

PSJ reports speakers fees from AstraZeneca, ProAdWise Communications; advisory board fees from AstraZeneca; and research funding from AstraZeneca, Boehringer Ingelheim, Analog Devices and Roche Diagnostics; and is a director of Global Clinical Trial Partners. PSJ's employer, the University of Glasgow, has been remunerated for clinical trial work from AstraZeneca, Bayer, Novartis and Novo Nordisk.

## PEER REVIEW

The peer review history for this article is available at https://www.webofscience.com/api/gateway/wos/peer-review/10.1111/dom.16388.
